# MiR-223 Regulates Human Embryonic Stem Cell Differentiation by Targeting the IGF-1R/Akt Signaling Pathway

**DOI:** 10.1371/journal.pone.0078769

**Published:** 2013-11-08

**Authors:** Yan-Hui Yu, Li Zhang, Deng-Shu Wu, Zheng Zhang, Fang-Fang Huang, Jian Zhang, Xiao-Ping Chen, De-Sheng Liang, Hui Zeng, Fang-Ping Chen

**Affiliations:** 1 Department of Hematology, Xiang-Ya Hospital, Central South University, Changsha, Hunan, China; 2 Department of Hematology, West China Hospital, Si Chuan University, Chengdu, Sichuan, China; 3 Department of Pharmacology, School of Pharmaceutical Sciences, Central South University, Changsha, Hunan, China; 4 Pharmacogenetics Research Institute, Institute of Clinical Pharmacology, Central South University, Changsha, Hunan, China; 5 State Key Laboratory of Medical Genetics of China, Central South University, Changsha, Hunan, China; Indiana University School of Medicine, United States of America

## Abstract

Currently, there are difficulties associated with the culturing of pluripotent human embryonic stem cells (hESCs), and knowledge regarding their regulatory mechanisms is limited. MicroRNAs (miRNAs) regulate gene expression and have critical functions in stem cell self-renewal and differentiation. Moreover, fibroblast growth factor (FGF) and the insulin-like growth factor receptor (IGF-1R) are key activators of signaling in hESCs. Based on the identification of complementary binding sites in miR-223 and *IGF-1R* mRNA, it is proposed that miR-223 acts as a local regulator of IGF-1R. Therefore, levels of miR-223 were detected in differentiated versus undifferentiated hESCs. In addition, proliferation, apoptosis, and differentiation were assayed in these two hESC populations and were compared in the presence of exogenous miR-223 and miR-223 inhibitor. Inhibition of miR-223 was found to maintain the undifferentiated state of hESCs, while addition of miR-223 induced differentiation. Furthermore, these effects were found to be likely dependent on IGF-1R/Akt signaling.

## Introduction

Human embryonic stem cells (hESCs) are derived from the totipotent cells of early embryos, and are distinguished by their capacity for unlimited self-renewal, undifferentiated proliferation *in vitro*, and their ability to differentiate into any cell type (pluripotency) [1]. Consequently, the application of hESCs in regenerative medicine holds great promise. It has been demonstrated that various factors regulate the differentiation of hESCs into different cell types. These include an array of transcription factors, such as Oct-4 and Nanog, which form self-regulatory networks to control a wide range of downstream genes [2], [3]. Several extrinsic signaling molecules and pathways have also been implicated in the maintenance of hESC pluripotency, including basic fibroblast growth factor (bFGF), insulin-like growth factor (IGF), and PI3K/Akt [4], [5]. Specifically, bFGF and IGF receptors have been found to contribute to the self-renewal capacity, poor differentiation, and enhanced proliferation of hESCs [6], [7]. In addition, the PI3K/Akt signaling pathway has been shown to mediate dynamic changes in the actin cytoskeleton, integrin signaling, and cell survival [8].

MicroRNAs (miRNAs) are small RNAs approximately 22 nucleotides in length that bind the 3′ untranslated region (3′-UTR) of messenger RNAs to post-transcriptionally regulate the translation of target genes involved in apoptosis, differentiation, and metabolic pathways [9]. Furthermore, accumulating evidence suggests that miRNAs play important roles in the development of stem cells, including the establishment of cellular identity and regulation of stem cell behavior [10]. Currently, miR-223 has been shown to have an essential role in the proliferation of granulocytes, gastric cancer cells, venous smooth muscle cells, and hematopoietic cells [11]. It is also a key factor during osteoclast differentiation [12]. However, the mechanisms by which miR-223 regulates the proliferation, apoptosis, and differentiation of hESCs remains unclear.

Using bioinformatics predictions, complementary binding sites have been identified in both miR-223 and the 3′-UTR of *IGF-1R* mRNA. Based on these data, we hypothesize that miR-223 acts as a local regulator of IGF-1R, thereby modulating development of hESCs. To further investigate this hypothesis, expression of miR-223 was monitored during hESC differentiation. In addition, two markers of pluripotency, Oct-4 and Nanog, were assayed in hESCs using lentiviral expression of miR-223 and a miR-223 inhibitor, in order to better understand the role and mechanistic details of miR-223 in the proliferation, apoptosis, and differentiation of hESCs.

## Materials and Methods

### Cell Maintenance and Differentiation

H1 and H9 human ES cell lines have been described previously [13] and were cultured on irradiated mouse embryonic fibroblasts (MEFs) in serum-free medium containing basic fibroblast growth factor (bFGF; R&D) [14]. Confluent cultures were harvested mechanically using a cell lifter, then were dissociated enzymatically using collagenase IV (Invitrogen). Cells were differentiated for five days in basic embryoid body medium containing DMEM/F12 (Gibco) supplemented with 20% knockout serum replacement (Gibco), 0.1 mM nonessential amino acids (Invitrogen), 0.1 mM β-mercaptoethanol (Sigma), 1 mM L-Glutamine (Invitrogen), 50 µg/ml ascorbic acid (Sigma), and 10 ng/ml bFGF (R&D). Cells were maintained at 37°C and 5% CO_2_, and were scored based on morphological criteria.

### Construction of pLV-THM-miR-223 and pLV-THM-miR-223-inhibitor Vectors

A third generation self-inactivating, lentivirus plasmid, pLV-THM (Addgene), was used as a transfer vector. Lentiviral vectors, psPAX2 and pMD2.G, were used as packaging and envelope plasmids, respectively. MiR-223 contains 20 nts and its mature sequence is: 5′-TGTCAGTTTGTCAAATACCCCA-3′. The mature sequence of the miR-223 inhibitor used was: 5′-UGGGGUAUUUGACAAACUCACA-3′, and it was chemically synthesized by Sangon Biotech Co. Ltd. (China). This oligonucleotide was inserted into the pLV-THM vector between the *MluI* and *ClaI* restriction sites according to the protocol [15], [16]. Lentiviral-based vectors containing pre-miR-223 or miR-223 inhibitor sequences were transformed into competent *Escherichia coli* cells and antibiotic-resistant colonies were selected on LB-ampicillin agar plates. Plasmids containing target genes were subsequently amplified, purified, and enzymatically digested. Plasmids were then subjected to polymerase chain reaction (PCR) assays, and clones with positive PCR results were subjected to DNA sequencing.

For transfection of plasmids, 293T cells were trypsinized and the cell density was adjusted to 1×10^6^ cells/ml with culture medium. Cells were then reseeded into 100 mm cell culture dishes and cultured for 24 h to achieve 90% confluence. Recombinant viral vectors encoding miR-223, the miR-223 inhibitor, and the two packaging plasmids were prepared using a plasmid extraction kit (Invitrogen). These plasmids were then transfected into 293T cells according to the manufacturer’s instructions. After 8 h, the medium was replaced with fresh complete medium. After 24 h, expression of GFP was assayed. After 48 h, the culture medium was collected and cellular debris was removed by centrifugation to obtain a high-titer lentivirus stock. Lentivirus without transgene was used as a negative control and was produced in the same manner.

### Virus Transduction and Detection of miR-223 Expression

Individual 60 mm cell culture dishes were plated with 2–3 hESC clones each in culture medium. After 24 h, cells were transduced with each lentivirus stock (5.0 × 10^5^ titer units), then were incubated for an additional 48–72 h prior to detection of miR-223 expression by quantitative real-time polymerase chain reaction (qRT-PCR). Small RNAs were isolated using a mirVana miRNA Isolation Kit (Ambion), and expression of miR-223 was detected using quantitative RT-PCR and Taqman miRNA assays (Applied Biosystems). Mature miR-223 was detected using a qRT-PCR miRNA Detection Kit and qRT-PCR Primer Sets, according to the manufacturer's instructions (Applied Biosystems). U6 small nuclear RNA was used as an internal control.

### Computational Identification of miRNAs Recognizing IGF-1R

Differentially expressed miRNAs were computationally screened using TargetScan Release 6.1 (http://www.targetscan.org) and MicroCosm Targets version 5 (http://www.ebi.ac.uk/enright-srv/microcosm) with IGF-1R (NM_000875) used as a target gene.

### Dual-luciferase Reporter Assay

To construct the dual luciferase vector, pmirGLO-IGF1R-3′-UTR, the 3′-UTR region of IGF-1R (NM_000875; 1–1240 bp) was PCR amplified using the following forward and reverse primers: 5′-CCCTCGAGGATCCTGAATCTGTGCAAAC-3′ (27 bp) and 5′-AGCGGCCGCCTTCCCAGCGAAATCATC -3′ (29 bp), respectively. The amplification product was then cloned into the vector, pmirGLO (Promega). As a control, the relative luciferase activity of a mutant IGF-1R-3′UTR reporter construct containing three mutated nucleotides within the putative seed sequence (226–228 bp) was used ***(*** ***Table 1*** ***)***. Cells (1 × 10^5^/well) were plated in 24-well plates, then incubated with 100 ng pmirGLO- IGF1R -3′UTR vector and 30 nM exogenous miR-223 or miR-223 inhibitor. Transfection of mutant IGF-1R-3′UTR (100 ng) was used as a control. Dual luciferase assays (Promega) were performed 48 h after transfection, and luminescence was measured using a BMG FLUOstar Optima (BMG Labtech GmbH). Statistical differences between treated and control cells were determined using Student’s *t*-test, with p<0.05.

**Table 1:** Potential interactions between miRNA-223 and the 3′UTR of IGF-1R.

**IGF1R-3′UTR 5′** …CCUGCCCAAACCCUUAACUGACA…|||||||**miRNA-223 3′** ACCCCAUAAACUGUUUGACUGU
**Mutant IGF1R-3′UTR 5′** …CCUGCCCAAACCCUUAAGACACA…||||**miRNA-223 3′** ACCCCAUAAACUGUUUGACUGU

### Detection of Oct-4 and Nanog Expression by qRT-PCR

Total RNA was extracted using Trizol reagent (Invitrogen) and reverse transcription was performed using a RT Kit according to the manufacturer’s protocol (Invitrogen). The primers used are listed. Oct-4: F 5′-CTTGGGCTACACAGGC-3′, R 5′-CTCAATACTCGTTCGCTTTC-3′; Nanog: F 5′-TTTGGAAGCTGCTGGGGAAG-3′, R 5′-ATGGGAGGAGGGGAGAGGA-3′; GAPDH: F 5′-GGAGCCAAAAGGGTCATC-3′, R 5′-CCAGTGAGTTTCCCGTTC-3′.

### Detection of Oct-4, Nanog, IGF-1R, and Akt Expression by Western Blot

Cells were lysed in RIPA lysis buffer supplemented with phenyl-methyl-sulphonyl fluoride (PMSF), 10 ul/ml sodium, and a protease inhibitor cocktail solution (all purchased from Santa Cruz Biotechnology). Cell lysates were separated by SDS/PAGE in 10% Tris-glycine gels and were transferred to nitrocellulose membranes. After blocking with 5% nonfat milk in TBS-T, the membranes were incubated with antibodies diluted 1∶500 in 5% BSA overnight at 4°C. The following antibodies were purchased from Cell Signal Technology: anti-Nanog, anti-Oct-4, anti-IGF-1R, as well as antibodies recognizing total Akt and phosphorylated Akt (p-Akt). After being washed, membranes were incubated with horseradish peroxidase (HRP)-conjugated secondary antibodies (Santa Cruz Biotechnology). Finally, all western blot exposures were within a linear range of detection and the intensities of the resulting bands were quantified using Quantity software available with the Che-mi Doc imaging system (Bio-Rad). Data are expressed as the mean ±sandard deviation (SD).

### Flow Cytometry

Cell cycle distribution and cell apoptosis were assayed using flow cytometry. Briefly, parental cells and stable subline cells were trypsinized, collected, washed, and stained. For cell cycle analysis, cells were stained with propidium iodide (PI, Sigma-Aldrich) and RNase A (Sigma) for 20 min at 4°C according to the manufacturer’s protocol (Sigma). For cell apoptosis, cells were stained with Annexin V-fluorescein isothiocyanate (FITC; BD) and PI for 10 min at 4°C according to the manufacturer’s protocol (BD Pharmingen).

### Statistical Analysis

Data are presented as the mean ± standard error of the mean (SEM). Student’s *t*-test was performed for statistical analyses using SPSS 17.0 software. A p-value less than 0.05 was considered statistically significant. All experiments were independently performed at least three times.

## Results

### MiR-223 Levels in Undifferentiated Versus Differentiated hESCs

**Figure 1:**
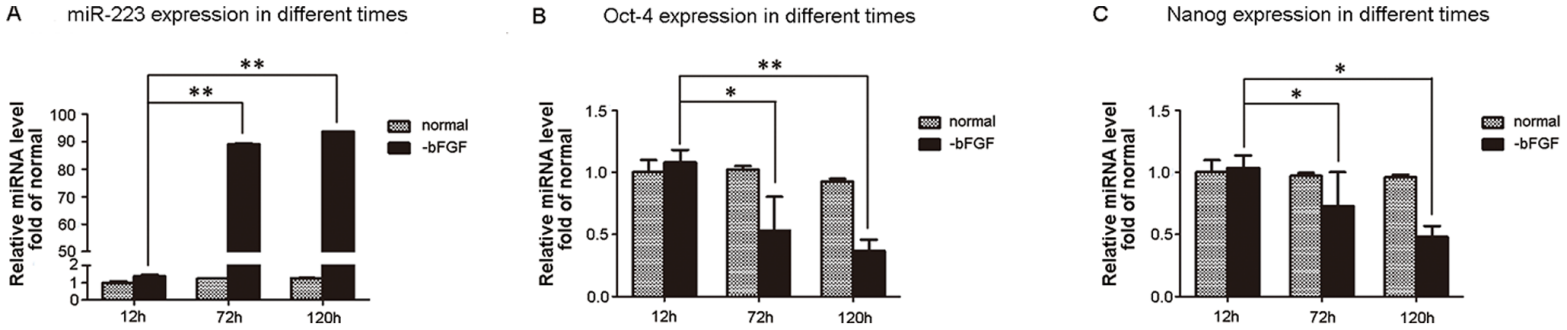
Levels of miR-223 detected in hESCs and spontaneously differentiated cells. **A,** Detection of miR-223 (**A**), Oct-4, (**B**), and Nanog (**C**) in hESCs and spontaneously differentiated cells 12, 72, and 120 h after withdrawal of bFGF (*p<0.05; **p<0.01; n = 6).

Using qRT-PCR, levels of miR-223 were detected in undifferentiated hESCs and spontaneously differentiated hESCs using H9 cells cultured in the presence and absence of bFGF, respectively (Fig. 1A). Expression levels of miR-223 were found to significantly increase in differentiated H9 cells. The pluripotency transcription markers, Oct-4 and Nanog, were also subsequently measured 12, 72, and 120 h after the withdrawal of bFGF. In contrast with miR-223, levels of Oct-4 and Nanog decreased in differentiated H9 cells (Fig. 1, B,C). Similar results were obtained for H1 cells (data not shown). These results suggest that miR-223 plays an important role in the differentiation of hESCs.

### MiR-223 Promotes Differentiation of hESCs

**Figure 2:**
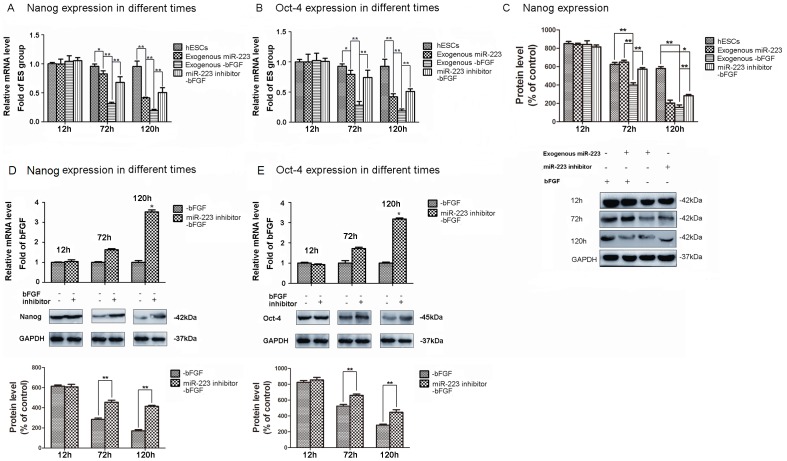
MiR-223 induces the differentiation of hESCs. Using qRT-PCR, Nanog (**A**) and Oct-4 (**B**) mRNA levels were assayed in control cells, cells expressing exogenous miR-223 with and without bFGF withdrawal, and cells expressing a miR-223 inhibitor that underwent withdrawal of bFGF for 12, 72, and 120 h (*p<0.05, **p<0.01; n = 6). (**C**) Levels of Nanog were detected by western blot 12, 72, and 120 h after withdrawal of bFGF for the groups. Levels of mRNA and protein were also assayed for Oct-4 (**D**) and Nanog (**E**) in the presence or absence of an miR-223 inhibitor 12, 72, and 120 h after withdrawal of bFGF (*p<0.05, **p<0.01; n = 6). For western blots, detection of GAPDH was used as a loading control and protein bands were semi-quantified using imaging analysis software. Data are expressed as the mean ± SD and are representative of the three independent experiments that were performed. *p<0.05, **p<0.01, n = 3.

To further investigate whether miR-223 can induce the differentiation of hESCs, H9 cells were transfected with lentiviruses expressing a mature miR-223 sequence or a miR-223 inhibitor, then were cultured with or without bFGF for 12, 72, and 120 h. When expression of Oct-4 and Nanog were assayed by qRT-PCR, mRNA levels of both transcription factors were observed to significantly decrease in cells expressing exogenous miR-223 following bFGF withdrawal compared to the levels of Oct-4 and Nanog detected in cells containing exogenous miR-223, cells expressing miR-223 inhibitor in the absence of bFGF, and in control cells (Fig. 2, A,B). Furthermore, western blotting confirmed the changes in Nanog expression detected with qRT-PCR (Fig. 2C). Taken together, these data indicate that miR-223 accelerates the differentiation of hESCs, while inhibition of miR-223 maintains the pluripotency of hESCs.

The spontaneous differentiation of H9 cells was further examined in the presence of a miR-223 inhibitor. Using qRT-PCR and western blotting, both mRNA and protein levels of Nanog and Oct-4 were found to be significantly upregulated following lentiviral expression of an miR-223 inhibitor and withdrawal of bFGF for 120 h, compared with withdrawal of bFGF in the absence of lentiviral infection (Fig. 2D, E). These data indicate that differentiation of hESCs was blocked, and similar results were observed with H1 cells (data not shown). In combination, these data suggest that miR-223 regulates hESCs differentiation.

### MiR-223 Regulates hESCs Differentiation likely by Targeting IGF-1R/Akt Signaling

**Figure 3:**
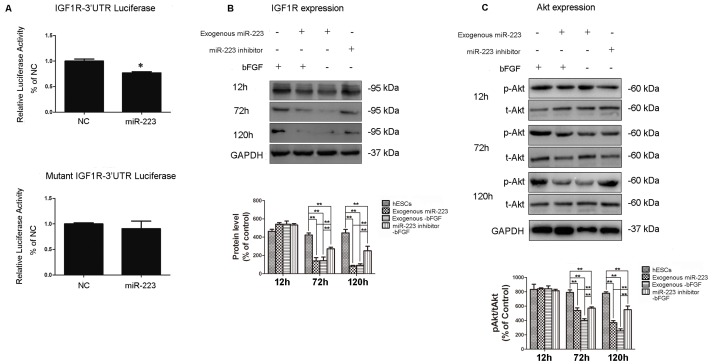
MiR-223 regulates hESCs differentiation likely via IGF-1R/Akt. (**A**) hESCs were infected with lentiviruses expressing miR-223 and wild type IGF-1R-3′UTR or a mutant IGF-1R-3′UTR construct. After 48 h, luciferase (Luc) activity was assayed. Data are presented as the mean ± SEM of the percentage of luciferase activity detected in control cells (*p<0.05; n = 4). Western blotting was performed to detect levels of IGF-1R (**B**) and phosphorylated Akt (p-Akt) and total Akt (t-Akt) (**C**) 12, 72, and 120 h after withdrawal of bFGF for the indicated groups. NC indicates the mock-vehicle group and GAPDH was used as a loading control. Protein bands were semi-quantified using imaging analysis software. Data are expressed as the mean ± SD and are representative of the three independent experiments that were performed. *p<0.05, **p<0.01, n = 3.

A dual luciferase assay was used to detect expression of IGF1R following the lentiviral infection of hESCs with miR-223 and the 3′UTR region of IGF-1R fused to a luciferase reporter. A decrease in luciferase activity was detected, indicating that IGF-1R can be regulated by miR-223. In contrast, the reporter activity of a construct containing a mutated version of IGF-1R-3′UTR was not affected by the presence of exogenous miR-223. These results suggest that IGF-1R may be a target of miR-223 (Fig. 3A). To confirm these observations, cell extracts were collected from control cells, cells expressing exogenous miR-223 with and without withdrawal of bFGF, and cells expressing a miR-223 inhibitor that underwent bFGF withdrawal. Western blotting detected a significant increase in levels of IGF-1R following expression of the miR-223 inhibitor compared to expression of exogenous miR-223 (Fig. 3B). Levels of Akt, a downstream kinase of the IGF-1R signaling pathway, were also examined in these four cell extracts. After 12 h, levels of phosphorylated Akt (p-Akt) were indistinguishable between the four groups. However, after 120 h, levels of p-Akt in both the control extracts and the extracts of cells expressing miR-223-inhibitor were significantly higher than those of cells expressing exogenous miR-223 (Fig. 3C). Similar results were observed for H1 cells in these assays (data not shown). Thus, miR-223 promotes hESCs differentiation likely via suppressing the IGF/Akt pathway.

### Effect of miR-223 on Cell-cycle Distribution and Apoptosis

**Figure 4:**
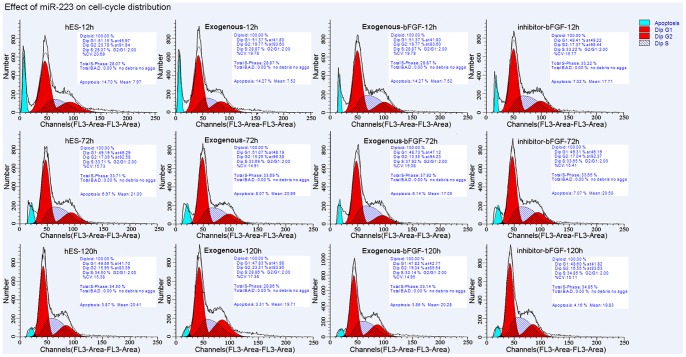
Effect of exogenous miR-223 on cell cycle progression. Cell cycle distribution for the indicated groups were assayed by flow cytometry 12(n = 3). Cells were treated with RNaseA and stained with PI. FACS analysis demonstrated that expression of exogenous miR-223, or an inhibitor of miR-223, does not affect cell cycle progression. The values shown are from experiments performed in triplicate ± SD.

**Figure 5:**
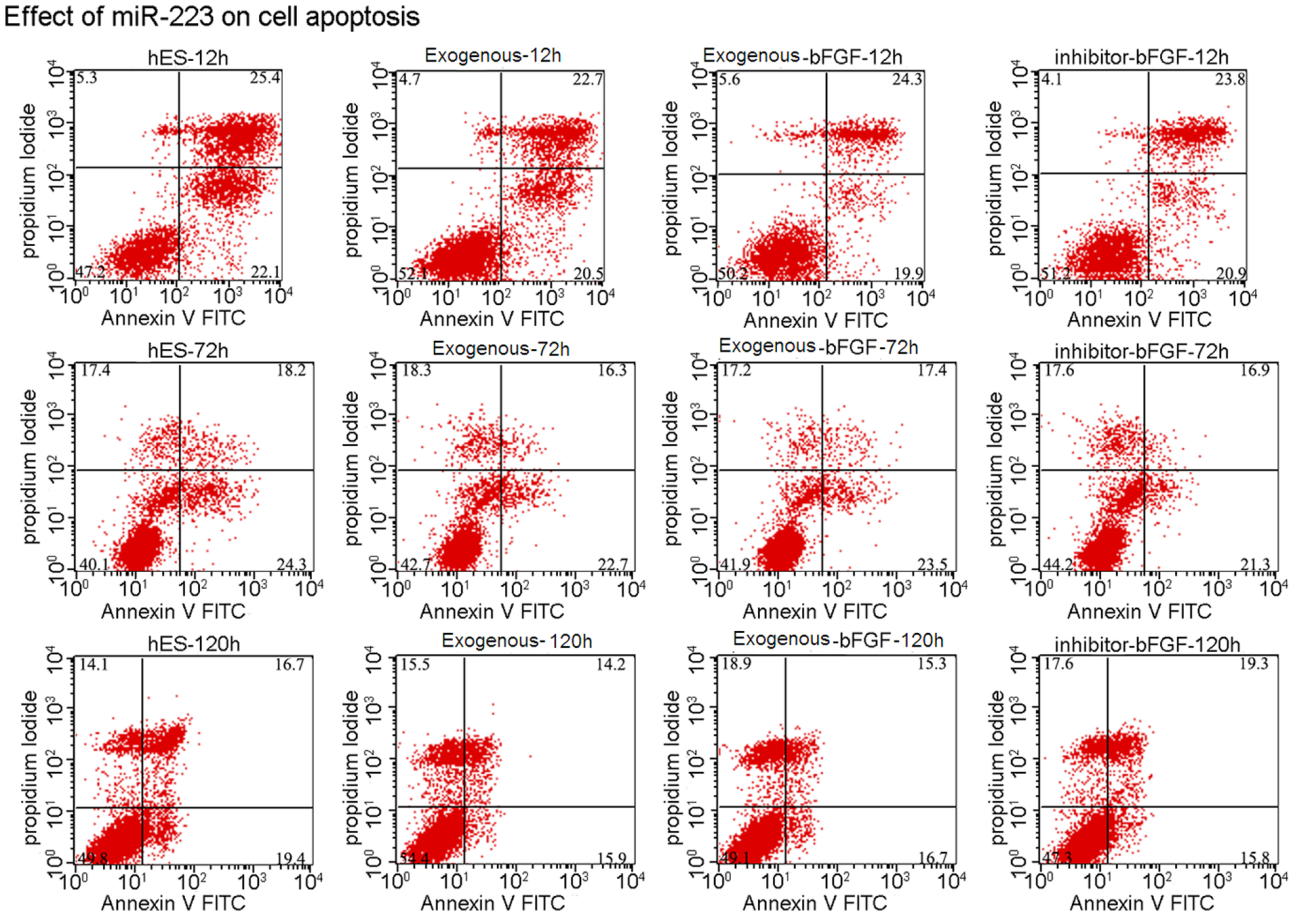
Effects of exogenous miR-223 on cell apoptosis. Cell apoptosis for the indicated groups were assayed by flow cytometry 12(n = 3). Cells were stained with AnnexinV and PI. FACS analysis shows that exogenous expression of miR-223, or an inhibitor of miR-223, does not affect cell apoptosis. The values shown are from experiments performed in triplicate ± SD.

To investigate the effects of miR-223 on cell cycle progression and apoptosis, control cells, cells expressing exogenous miR-223 with and without withdrawal of bFGF, and cells expressing miR-223 inhibitor that underwent withdrawal of bFGF were assayed by flow cytometry following staining with PI. No significant difference in the percentage of cells distributed in the G_0_/G_1_ phase and the S and G_2_/M phases was observed (Fig. 4). Furthermore, no significant difference in the percentage of apoptotic cells detected following staining with AnnexinV and PI was observed for the four groups (Fig. 5). Parallel assays conducted using H1 cells also obtained similar data (data not shown). Taken together, these results suggest that miR-223 is not required for the proliferation of hESCs, and this is inconsistent with the proproliferative role that miR-223 has exhibited in other cell types [17].

## Discussion

Although Oct-4 and Nanog are recognized as specific markers of undifferentiated pluripotency [18], little information is available regarding post-transcriptional mechanisms that regulate the pluripotency, self-renewal, and early determination of hESC fate. However, several miRNAs have been found to play roles in the proliferation, apoptosis, and differentiation of hESCs. Furthermore, recent evidence suggests that miR-223, an evolutionarily conserved miRNA, may represent a potential biomarker for recurrent ovarian cancer and sepsis [19]. Thus, the role of miR-223 in the proliferation, apoptosis, and differentiation of hESCs was investigated.

In undifferentiated hESCs, low levels of miR-223 were detected. In contrast, differentiated hESCs expressed high levels of miR-223. Based on these results, it is hypothesized that miR-223 plays a key role in the differentiation of hESCs. To test this hypothesis, lentiviral transduction was performed to achieve overexpression of miR-223 and miR-223-targeted inhibition. While the former aggravated hESC differentiation, the latter was able to block differentiation to a certain extent. Notably, however, neither overexpression, nor inhibition, of miR-223 exerted significant effects on cell proliferation and apoptosis. Collectively, these results suggest that miR-223 enhances the differentiation of hESCs without affecting the processes of proliferation and apoptosis, and this is in contrast with the role of miR-223 in osteoclasts where miR-223 has been shown to inhibit both cell differentiation and proliferation [12], [20]. Furthermore, in SW480 cells and rat aortic smooth muscle cells, Wang et al. previously reported that miR-223 suppressed cell proliferation by targeting IGF-1R [21]. Although these results differ from those of the present study, this may be due to cell type-specific differences in the biological characteristics of hESCs.

IGF-1R is also critical for the self-renewal of hESCs [22], [23]. In the present study, IGF-1R was found to be a target gene of miR-223 based on several lines of evidence. First, the computational screening programs, TargetScan and MicroCosm, identified IGF-1R as a target gene. Correspondingly, overexpression of miR-223 resulted in a significant decrease in luciferase activity for the IGF-1R- 3′UTR reporter. Second, cells expressing a mutant version of IGF-1R did not respond to miR-223. Third, IGF-1R was significantly downregulated following overexpression of miR-223. Based on these data, it appears that the differentiation of hESCs is regulated by miR-223 via IGF-1R.

IGF-1R can also activate many downstream kinases, including Akt, Erk, and Ampk [24]. Furthermore, Akt plays an important role in maintaining the pluripotent state of hESCs, and lies downstream of bFGF signaling [7], [25], [26]. In the present study, an increase in levels of p-Akt were detected in hESCs in the presence of a miR-223 inhibitor, thereby suggesting that miR-223 regulates the differentiation of hESCs via the IGF/Akt pathway.

Based on the results of the present study, it appears that miR-223 regulates the differentiation of hESCs by targeting IGF-1R and its downstream Akt signaling pathway. It has been demonstrated that treating hESCs with Akt inhibitor (like LY294002) induces hESCs differentiation [27], [28]. Our current study revealed that miR-223 behaves as a Akt inhibitor during hESCs differentiation. Combined with aforementioned evidence from previous reports, our data suggested that miR-223 promotes hESCs differentiation likely via suppressing the IGF/Akt pathway. Moreover, although the miR-223 gene is located on the X chromosome, similar results were obtained for both H9 and H1 cell lines, which were originally isolated from female and male individuals, respectively. Therefore, this novel mechanism by which hESC differentiation is regulated by miR-223 warrants further investigation, and has the potential to facilitate the use of hESCs in transplantation studies and drug screening assays.

## References

[1] Wilmut I, West MD, Lanza RP, Gearhart JD, Smith A, et al (2005) Human embryonic stem cells. Science 310: 1903. 10.1126/science.1123832 16352868

[2] Boyer LA, Lee TI, Cole MF, Johnstone SE, Levine SS, et al (2005) Core transcriptional regulatory circuitry in human embryonic stem cells. Cell 122: 947–956. 16153702 10.1016/j.cell.2005.08.020 PMC3006442

[3] Jaenisch R, Young R (2008) Stem cells, the molecular circuitry of pluripotency and nuclear reprogramming. Cell 132: 567–582. 18295576 10.1016/j.cell.2008.01.015 PMC4142810

[4] Pera MF, Tam PP (2010) Extrinsic regulation of pluripotent stem cells. Nature 465: 713–720. 20535200 10.1038/nature09228

[5] Chen YG, Li Z, Wang XF (2012) Where PI3K/Akt meets Smads: the crosstalk determines human embryonic stem cell fate. Cell Stem Cell 10: 231–232. 22385648 10.1016/j.stem.2012.02.008 PMC3586792

[6] McDevitt TC, Laflamme MA, Murry CE (2005) Proliferation of cardiomyocytes derived from human embryonic stem cells is mediated via the IGF/PI 3-kinase/Akt signaling pathway. J Mol Cell Cardiol 39: 865–873. 16242146 10.1016/j.yjmcc.2005.09.007 PMC3505759

[7] Eiselleova L, Matulka K, Kriz V, Kunova M, Schmidtova Z, et al (2009) A complex role for FGF-2 in self-renewal, survival, and adhesion of human embryonic stem cells. Stem Cells 27: 1847–1857. 19544431 10.1002/stem.128 PMC2798073

[8] Rodriguez-Viciana P, Warne PH, Khwaja A, Marte BM, Pappin D, et al (1997) Role of phosphoinositide 3-OH kinase in cell transformation and control of the actin cytoskeleton by Ras. Cell 89: 457–467. 9150145 10.1016/s0092-8674(00)80226-3

[9] Lewis BP, Burge CB, Bartel DP (2005) Conserved seed pairing, often flanked by adenosines, indicates that thousands of human genes are microRNA targets. Cell 120: 15–20. 15652477 10.1016/j.cell.2004.12.035

[10] Gangaraju VK, Lin H (2009) MicroRNAs: key regulators of stem cells. Nat Rev Mol Cell Biol 10: 116–125. 19165214 10.1038/nrm2621 PMC4118578

[11] Johnnidis JB, Harris MH, Wheeler RT, Stehling-Sun S, Lam MH, et al (2008) Regulation of progenitor cell proliferation and granulocyte function by microRNA-223. Nature 451: 1125–1129. 18278031 10.1038/nature06607

[12] Sugatani T, Hruska KA (2007) MicroRNA-223 is a key factor in osteoclast differentiation. J Cell Biochem 101: 996–999. 17471500 10.1002/jcb.21335

[13] Ahn SE, Kim S, Park KH, Moon SH, Lee HJ, et al (2006) Primary bone-derived cells induce osteogenic differentiation without exogenous factors in human embryonic stem cells. Biochem Biophys Res Commun 340: 403–408. 16389066 10.1016/j.bbrc.2005.12.020

[14] Xu C, Inokuma MS, Denham J, Golds K, Kundu P, et al (2001) Feeder-free growth of undifferentiated human embryonic stem cells. Nat Biotechnol 19: 971–974. 11581665 10.1038/nbt1001-971

[15] Ebert MS, Neilson JR, Sharp PA (2007) MicroRNA sponges: competitive inhibitors of small RNAs in mammalian cells. Nat Methods 4: 721–726. 17694064 10.1038/nmeth1079 PMC3857099

[16] Shin KJ, Wall EA, Zavzavadjian JR, Santat LA, Liu J, et al (2006) A single lentiviral vector platform for microRNA-based conditional RNA interference and coordinated transgene expression. Proc Natl Acad Sci U S A 103: 13759–13764. 16945906 10.1073/pnas.0606179103 PMC1557799

[17] Song L, Duan P, Guo P, Li D, Li S, et al (2012) Downregulation of miR-223 and miR-153 mediates mechanical stretch-stimulated proliferation of venous smooth muscle cells via activation of the insulin-like growth factor-1 receptor. Arch Biochem Biophys 528: 204–211. 23046980 10.1016/j.abb.2012.08.015

[18] Chambers I, Colby D, Robertson M, Nichols J, Lee S, et al (2003) Functional expression cloning of Nanog, a pluripotency sustaining factor in embryonic stem cells. Cell 113: 643–655. 12787505 10.1016/s0092-8674(03)00392-1

[19] Wang JF, Yu ML, Yu G, Bian JJ, Deng XM, et al (2010) Serum miR-146a and miR-223 as potential new biomarkers for sepsis. Biochem Biophys Res Commun 394: 184–188. 20188071 10.1016/j.bbrc.2010.02.145

[20] Jia CY, Li HH, Zhu XC, Dong YW, Fu D, et al (2011) MiR-223 suppresses cell proliferation by targeting IGF-1R. PLoS One 6: e27008. 22073238 10.1371/journal.pone.0027008 PMC3206888

[21] Standley PR, Obards TJ, Martina CL (1999) Cyclic stretch regulates autocrine IGF-I in vascular smooth muscle cells: implications in vascular hyperplasia. Am J Physiol 276: E697–705. 10198306 10.1152/ajpendo.1999.276.4.E697

[22] Wang L, Schulz TC, Sherrer ES, Dauphin DS, Shin S, et al (2007) Self-renewal of human embryonic stem cells requires insulin-like growth factor-1 receptor and ERBB2 receptor signaling. Blood 110: 4111–4119. 17761519 10.1182/blood-2007-03-082586 PMC2190616

[23] Freund C, Ward-van Oostwaard D, Monshouwer-Kloots J, van den Brink S, van Rooijen M, et al (2008) Insulin redirects differentiation from cardiogenic mesoderm and endoderm to neuroectoderm in differentiating human embryonic stem cells. Stem Cells 26: 724–733. 18096723 10.1634/stemcells.2007-0617

[24] Estan MC, Calvino E, de Blas E, Boyano-Adanez Mdel C, Mena ML, et al (2012) 2-Deoxy-D-glucose cooperates with arsenic trioxide to induce apoptosis in leukemia cells: involvement of IGF-1R-regulated Akt/mTOR, MEK/ERK and LKB-1/AMPK signaling pathways. Biochem Pharmacol 84: 1604–1616. 23041229 10.1016/j.bcp.2012.09.022

[25] Kim SJ, Cheon SH, Yoo SJ, Kwon J, Park JH, et al (2006) Retraction notice to “Contribution of the PI3K/Akt/PKB signal pathway to maintenance of self-renewal in human embryonic stem cells” [FEBS Lett. 579 (2005) 534–540]. FEBS Lett 580: 1529. 16521304 10.1016/j.febslet.2006.01.068

[26] Miyagawa Y, Okita H, Hiroyama M, Sakamoto R, Kobayashi M, et al (2011) A microfabricated scaffold induces the spheroid formation of human bone marrow-derived mesenchymal progenitor cells and promotes efficient adipogenic differentiation. Tissue Eng Part A 17: 513–521. 20818998 10.1089/ten.TEA.2009.0810

[27] McLean AB, D′Amour KA, Jones KL, Krishnamoorthy M, Kulik MJ, et al (2007) Activin a efficiently specifies definitive endoderm from human embryonic stem cells only when phosphatidylinositol 3-kinase signaling is suppressed. Stem Cells 25: 29–38. 17204604 10.1634/stemcells.2006-0219

[28] Singh AM, Reynolds D, Cliff T, Ohtsuka S, Mathyses A, et al (2012) Signaling network crosstalk in human pluripotent cells: a Smad2/3-regulated switch that controls the balance between self-renewal and differentiation. Cell Stem Cell 10(3): 312–326. 22385658 10.1016/j.stem.2012.01.014 PMC3294294

